# Trans-activation, post-transcriptional maturation, and induction of antibodies to HERV-K (HML-2) envelope transmembrane protein in HIV-1 infection

**DOI:** 10.1186/1742-4690-11-10

**Published:** 2014-01-28

**Authors:** Henri-Alexandre Michaud, Miguel de Mulder, Devi SenGupta, Steven G Deeks, Jeffrey N Martin, Christopher D Pilcher, Frederick M Hecht, Jonah B Sacha, Douglas F Nixon

**Affiliations:** 1 Division of Experimental Medicine, Department of Medicine, University of California, San Francisco, USA; 2 HIV/AIDS Program, Department of Medicine, University of California, San Francisco, USA; 3 Department of Epidemiology and Biostatistics, University of California, San Francisco, USA; 4 Vaccine & Gene Therapy Institute, Oregon Health & Science University, Beaverton, OR 97006, USA; 5 Oregon National Primate Research Center, Oregon Health & Science University, Beaverton, OR 97006, USA; 6 Microbiology, Immunology & Tropical Medicine, School of Medicine & Health Sciences, The George Washington University, Ross Hall 502, 2300 Eye Street, NW, Washington, DC 20037, USA

**Keywords:** HIV, Antibody, HERV, Endogenous retroviruses, Transmembrane, Envelope, Elite controllers, Alternative transcripts

## Abstract

**Background:**

Human Endogenous Retroviruses (HERVs) comprise about 8% of the human genome and have lost their ability to replicate or to produce infectious particles after having accumulated mutations over time. We assessed the kinetics of expression of HERV-K (HML-2) Envelope mRNA transcript and surface unit (SU) and transmembrane (TM) subunit proteins during HIV-1 infection. We also mapped the specificity of the humoral response to HERV-K (HML-2) Envelope protein in HIV-1 infected subjects at different stages of disease, and correlated the response with plasma viral load.

**Results:**

We found that HIV-1 modified HERV-K (HML-2) Env mRNA expression, resulting in the expression of a fully N-glycosylated HERV-K (HML-2) envelope protein on the cell surface. Serological mapping of HERV-K (HML-2) envelope protein linear epitopes revealed two major immunogenic domains, one on SU and another on the ectodomain of TM. The titers of HERV-K (HML-2) TM antibodies were dramatically increased in HIV-1 infected subjects (p < 0.0001). HIV-1 infected adults who control HIV-1 in the absence of therapy (“elite” controllers) had a higher titer response against TM compared to antiretroviral-treated adults (p < 0.0001) and uninfected adults (p < 0.0001).

**Conclusions:**

These data collectively suggest that HIV-1 infection induces fully glycosylated HERV-K (HML-2) envelope TM protein to which antibodies are induced. These anti-HERV-K (HML-2) TM antibodies are a potential marker of HIV-1 infection, and are at higher titer in elite controllers. HERV-K (HML-2) envelope TM protein may be a new therapeutic target in HIV-1 infection.

## Background

Human endogenous retroviruses (HERVs) comprise about 8% of the human genome [1]. Their ability to replicate or produce infectious particles is inhibited by host restriction [2,3] and they are now considered to be stably integrated, largely silent, and transmitted in a Mendelian fashion [4]. However, HERV-K (HML-2), which is the most recently integrated of the HERV families, exhibits more ability to express proteins than other older HERV families [5,6]. The genome of HERV-K (HML-2), the gag, pol, pro, and env genes, are flanked by two Long Terminal Repeats (LTR,) and it is possible to express all the viral proteins under specific conditions [2,7]. HERV expression has been associated with autoimmune diseases [8-13] and cancers [14-19], and in these settings mRNA transcripts and proteins are found in blood or tissues. Despite their status as self-antigens, translated HERV products can induce an immune response that correlates with disease progression or regression in some cancers [20-25].

During HIV-1 infection we have previously shown reactivation of HERV-K (HML-2) [26,27]. The mechanisms leading to HERV-K (HML-2) expression are still being elucidated but HIV-1 Vif and Tat protein have been implicated [27,28]. These studies strengthen the concept that HIV-1 specifically induces the transcription of HERV-K (HML-2) mRNA which results in the expression of HERV-K (HML-2) proteins in HIV-1 infected cells. We have shown that an anti-HERV-K (HML-2) cellular immune response is generated in HIV-1 infected patients (HIV^pos^), significantly increased in elite controllers, and HERV-K specific T-cell clones can eliminate HIV-1 infected cells *in vitro*[26,27,29]. Whether HERV-K reactivation in HIV-1 infection leads to an anti-HERV-K (HML-2) antibody response is controversial [30].

The goal of this study was to identify B-cell epitopes present on the HERV-K (HML-2) Env protein, and ascertain how HIV-1 infection impacts anti-HERV humoral immunity. HERV-K (HML-2) Env is composed of three proteins, the signal peptide (SP), the surface unit (SU) and the transmembrane (TM) protein [31]. We identified two major immunogenic domains of HERV-K (HML-2) Env, one on SU and another on the ectodomain of TM. We found that HIV-1 preferentially modified the anti-HERV-K HML-2 TM antibody response. While the anti-SU antibody titer was largely unchanged between HIV-1 infected (HIV^pos^) or uninfected subjects (HIV^neg^), the titers of anti-TM antibodies were dramatically increased in HIV^pos^. Although the anti-HERV-K (HML-2) TM response correlated with HIV-1 plasma viral load in viremic non-controllers and was reduced during efficient HAART treatment, elite controllers who naturally suppress HIV-1 viremia have a higher titer antibody response against HERV-K (HML-2) TM compared to HIV^neg^ or HAART-suppressed patients. We determined that HIV-1 infection modifies HERV-K (HML-2) Env mRNA expression, which leads to a fully N-glycosylated HERV-K (HML-2) transmembrane envelope protein on the cell surface. These data are consistent with our overall conceptual model that HIV-1 infection changes HERV-K (HML-2) expression and protein production within an infected cell.

## Results

### Identification of two linear antibody epitopes in HERV-K (HML-2) Envelope

**Figure 1:**
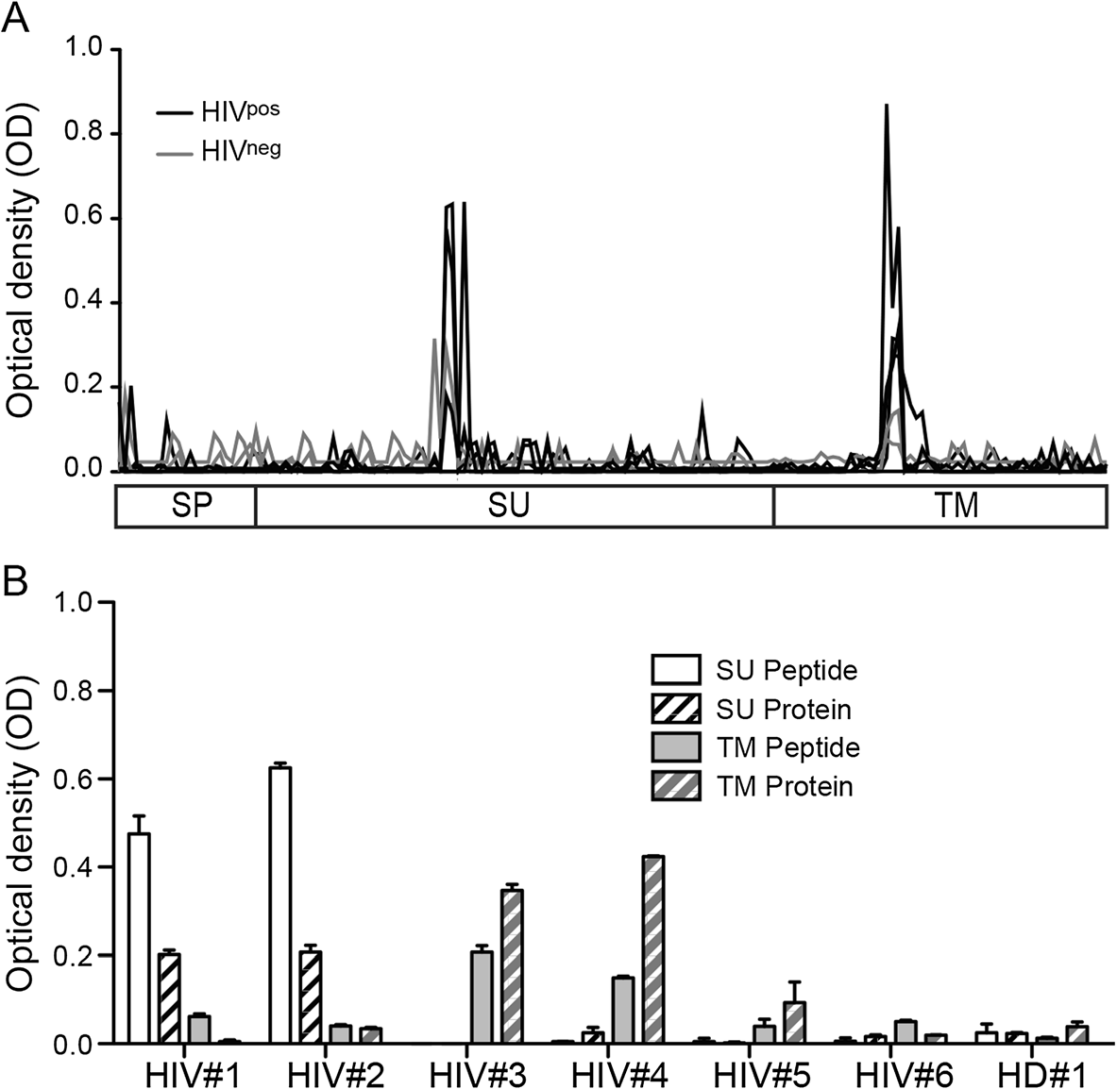
**Identification of two linear epitopes on HERV-K (HML-2) Env. (A)** 4 sera from chronically HIV-1 infected patients (HIV^pos^; black) and 2 sera from seronegative low risk healthy donors (HIV^neg^; grey) were used for antibody epitope identification by ELISA. The 3 sub-units signal peptide (SP), surface-unit protein (SU) and trans-membrane proteins (TM) are represented by 172 redundant 15mers. The lines represent the average of duplicate signals (OD) for each individual. **(B)** Sera from patients have antibodies reacting only with the SU-peptide (HIV#1 and #2), only with the TM-peptide (HIV#3 and #4), or negative for both epitopes (HIV#5, #6 and HIV^neg^ #1), were used to confirm the signal obtained with the peptide-based ELISA. Plain columns represent the peptides and the hatched columns represent the recombinant protein; SU in white and TM in gray. The ELISA was performed in duplicate, and the error bars represent the SEM.

To identify the immunogenic domains on HERV-K (HML-2) Env, we first mapped humoral linear epitopes on the HERV-K (HML-2) Env protein using a set of 172 “15mer” HERV-K (HML-2) Env peptides, in a peptide-based ELISA assay. We used sera from four HIV-1 infected subjects (HIV^pos^) and two HIV-1 seronegative low risk healthy donors (HIV^neg^). We found two strong immunogenic domains, one with homology to a domain previously described on the HERV-K (HML-2) Env SU protein [9] (see Discussion), and one novel epitope on TM (Figure 1A). We then compared the responses using recombinant HERV SU or TM proteins to individual peptides and observed concordance. Using HERV-K (HML-2) SU or TM recombinant proteins, and sera from HIV-1 positive or negative subjects, we observed a concordance of responsiveness between recombinant protein and peptide (Figure 1B). We confirmed that the humoral response directed against HERV-K (HML-2) Env was restricted to two linear epitopes, one on the SU protein and the other to a novel epitope on the TM protein, in both HIV^neg^ and HIV^pos^ subjects.

### Comparison of the HERV-K (HML-2) envelope SU and TM humoral responses in healthy donors and HIV-1-infected subjects

**Figure 2:**
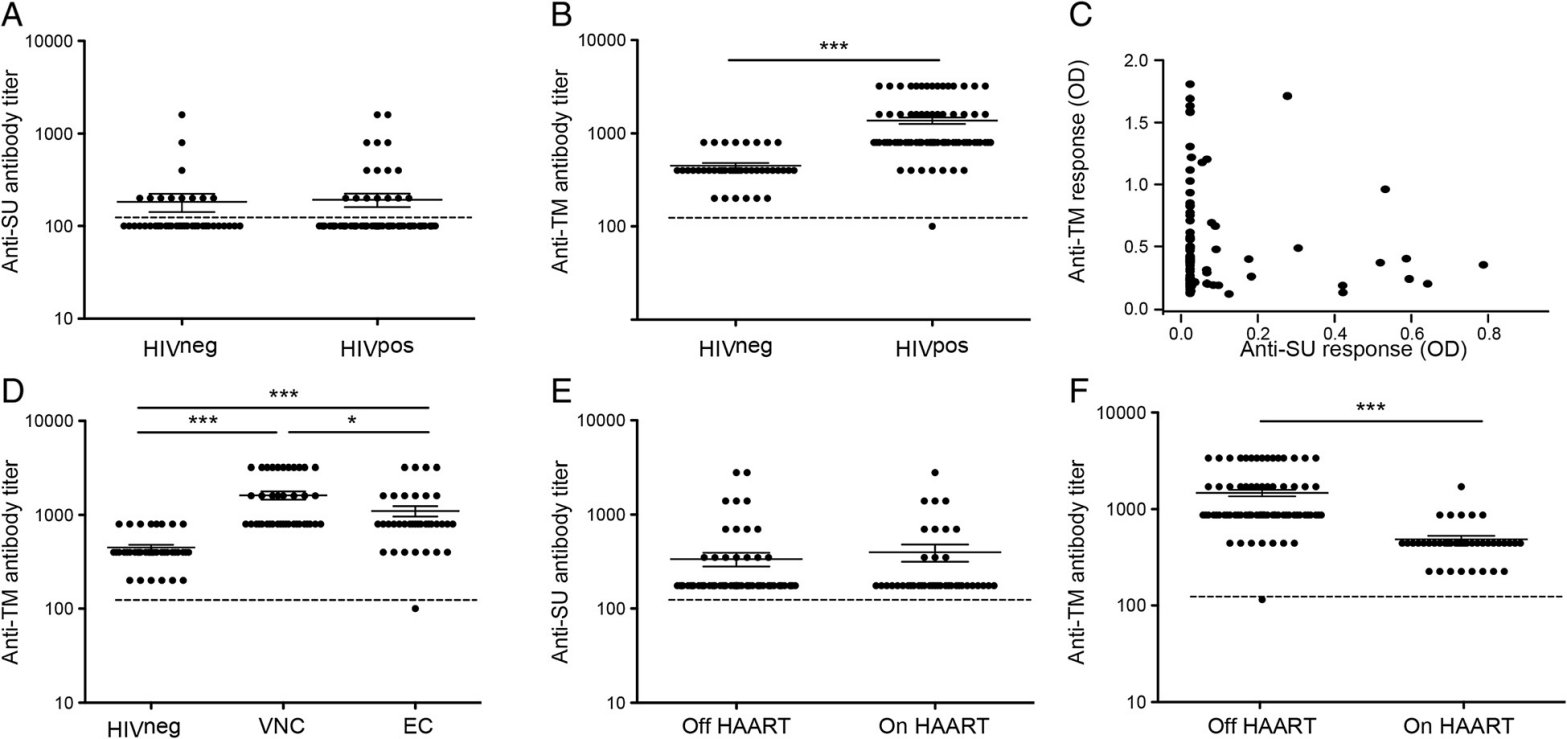
**Humoral response against HERV-K Env TM and SU.** The detection of antibodies was performed for 40 seronegative low risk healthy donors (HIV^neg^) and 80 chronic HIV-1 subjects (HIV^pos^), 40 elite controllers (EC) and 40 viremic-non-controllers (VNC). Dashed bar represents the threshold detection (1:200 dilution). Sera with a negative signal at 1:200 dilution were considered negative, and plotted below the dashed bar. **A)** HIV^neg^ or HIV^pos^ showed no difference for the anti-SU response, with a mean titer of 180 and 190, respectively. **B)** HIV^pos^ showed an increase of anti-TM antibody compared to HIV^neg^, with a mean titer of 1350 and 450, respectively. **C)** Plots represent the two antibody responses (SU and TM) for one HIV^pos^ patient. Detection was done on the same plate with sera diluted at 1:200 and 1:400 for SU and TM, respectively, n = 80. **(D)** VNC had the highest anti-TM titer (1600) compared to HIV^neg^ (450) and EC (1100). EC had a significant higher anti-TM titer compared to HIV^neg^. E and F) Comparison of the anti-SU **(E)**, or the anti-TM **(F)**, titers between HIV^pos^ patients on or off HAART. On HAART n = 40; Off HAART n = 80. Detection of anti-SU antibodies. ODs were normalized with serum from a high responder. The STDEV intra experiment was less than 7%. Detection of anti-TM antibodies. Sera were used at 1:400. OD were normalized with serum from a high responder. The STDEV intra experiment was less than 4%. The statistical significance of between the different groups was established using the Mann Whitney *T* test for **A**, **B**, **E** and **F**, and a Kruskal-Wallis and Dunn’s Multiple Comparison Test for D. The figure shows the representative results of three independent experiments. A p value <0.05 was considered as significant. *p < 0.05, **p < 0.01, ***p < 0.001.

Using the two amino-acid sequences identified, we performed a serological screen of HERV-K (HML-2) Env responses in 80 chronically infected, untreated HIV-1-infected subjects (HIV^pos^) and 40 HIV^neg^ subjects, in a cross sectional study (subject characteristics are detailed in Table 1). No differences in titer for anti-HERV-K (HML-2) SU responses between HIV^pos^ and HIV^neg^ were observed, with a mean of 1:180 and 1:190 respectively (Figure 2A). However, compared to uninfected HIV^neg^, the antibody response to HERV-K (HML-2) TM was significantly increased in HIV^pos^ subjects (p < 0.0001), with a mean of titer of 1:450 and 1:1370 respectively (Figure 2B). A comparison of within each HIV^pos^ subject anti-HERV-K (HML-2) TM and -SU responses indicated an exclusivity of response specificity, as those making anti-HERV-K (HML-2) TM responses in general did not make anti-HERV-K (HML-2) SU responses and vice versa (Figure 2C).

**Table 1:** Characteristics of study subjects

**Participant category (n)**	**Median age (yr [IQR])**	**Gender**^**a**^		**Median CD4+T cell count (cells/mm3 [IQR])**	**Median HIV-1 viral load (copy/ml [IQR]**
		**M**	**F**		
Elite controllers (40)	50 [44.25-55.75]	24	15	771 [500-1108]	<50
HAART suppressor (40)	50.50 [43.75-54.75]	31	8^*^	609 [452-807]	<50
Viremic non controllers (40)	39.50 [32.25-49]	35	3^*+^	444 [362-625]	40,474 [21,322-83,318]
HIV-1 negative (80)^b^	18-30 (18)	40	40	n/a	
	31-50 (24)				
	51-70 (34)				
	>70 (4)				

^a^F, female; M, male.

^b^Some information about the CD4+ T-cell count was not available (n/a). Only a range of age was available.

^*^One patient in this cohort was transgender male to female.

^+^One patient in this cohort was intersex.

**Figure 3:**
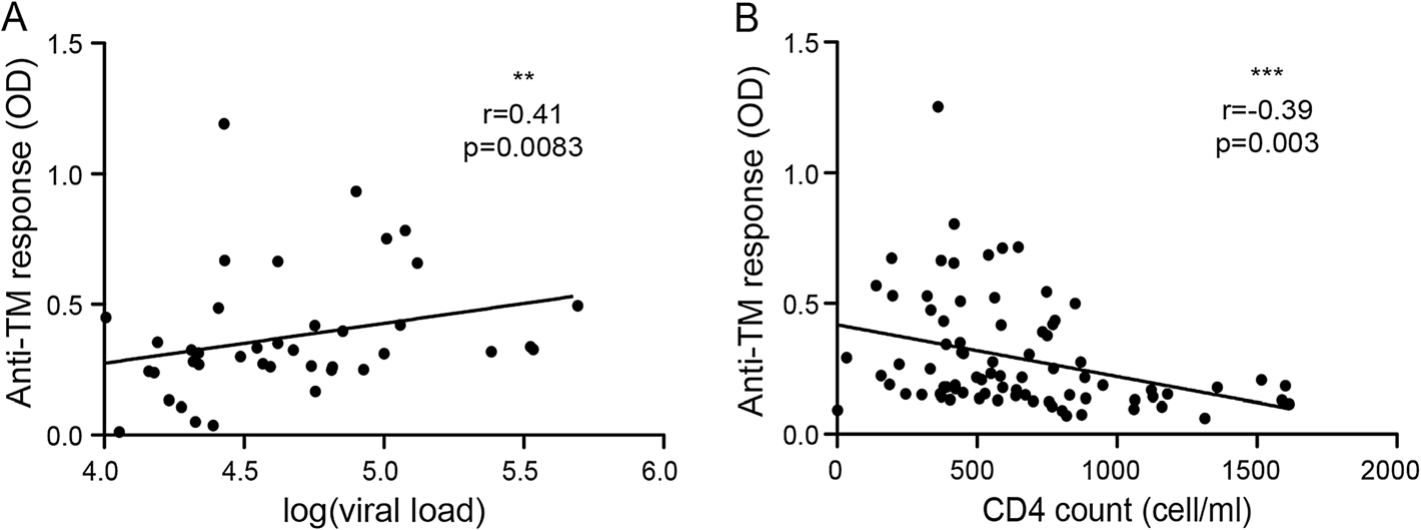
**Anti-TM response correlates with HIV-1 activity. A)** The viremia for 40 VNC was measured by real time PCR and expressed as log of copy/ml of blood. **B)** The anti-TM response for 80 HIV^pos^ untreated patients was inversely correlated to the CD4+ T cell count (cells/mm3). The detection was done on the same plate with sera diluted at 1:800. The statistical analysis was performed with the Spearman test, or with a Mann & Whitney *T* test, with a p value <0.05 considered as significant. The figure shows the representative results of three independent experiments. **p < 0.01, ***p < 0.001.

To determine whether any relationship existed between anti-HERV-K (HML-2) Env antibody responses and clinical progression, we assessed responses among 40 elite controllers and 40 viremic non-controllers. Although no significant difference was detected for the anti-HERV-K (HML-2) SU response (data not shown), non-controllers had a significantly greater titer of anti-HERV-K (HML-2) TM antibodies (mean 1:1600) compared to controllers (mean 1:1100). Both controllers and non-controllers had a greater anti-HERV-K (HML-2) TM response compared to the HIV^neg^ subjects (mean 1:450) (Figure 2D). We then examined responses during antiretroviral treatment, and found that treatment did not significantly modify the anti-HERV-K (HML-2) SU titer (mean 1:190 off-HAART versus 1:230 on-HAART). However, successful treatment was associated with a decrease in the anti-HERV-K (HML-2) TM response (mean 1:1370 off-HAART versus 1:440 on-HAART), to a titer similar to that found in seronegative healthy donors (Figure 2E,F). We examined the potential relationships between responses, time from infection, plasma viremia and CD4+ T cell count. While there was no correlation between the anti-HERV-K (HML-2) SU response and plasma viremia or CD4+ T cell count, there was a direct correlation between the anti-HERV-K (HML-2) TM humoral response and the plasma viral load (p = 0.0083) (Figure 3A). Furthermore, there was an inverse correlation between the anti-HERV-K (HML-2) TM humoral response and CD4+ T cell count (p = 0.003) (Figure 3B). These cross sectional data, from 120 HIV^pos^ subjects, showed that only the response against HERV-K HML-2 TM is modified during HIV-1 infection. Although the anti-HERV-K (HML-2) TM response correlated with the presence or absence of HIV-1 viremia in non-controllers, HAART suppressed and HIV^neg^ subjects, a strong anti-HERV-K (HML-2) TM response was still detected in elite controllers, despite the absence of detectable viremia.

### Detection of HERV-K (HML-2) envelope specific B-cells

**Figure 4:**
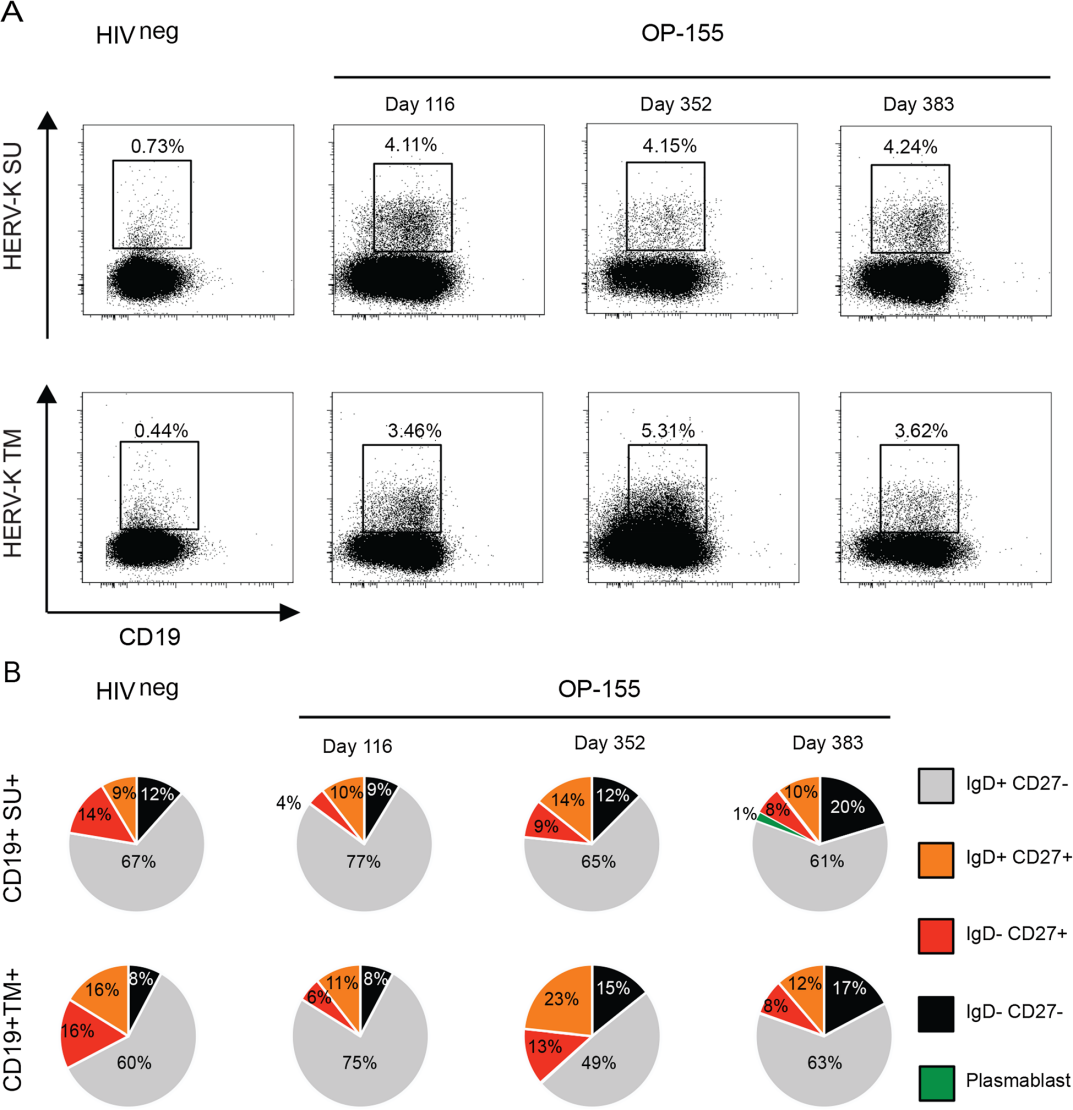
**Anti-HERV-K (HML-2) TM and SU specific B-cell responses. A)** The presence of HERV-K specific B-cells was detected at three time points from the same patient OP-115. CD19+ gated plots show the double population CD19 + tetramer + and the graphics represent the specific B-cells sub-populations. **B)** The study of the B-cell subset was based on CD27 and IgD extracellular expression. Memory cells (CD27+) were identified by their IgD expression; CD27+ IgD+: unswitched memory; CD27+ IgD-: switched memory. A Plasmablast is defined by the absence of IgD expression and a high expression of CD27.

To assess the presence and frequency of HERV-K (HML-2) Env specific B-cells in one healthy donor and one HIV-1 infected subject (OP-115), we used SU and TM epitope-specific peptides conjugated to biotin in a flow cytometry assay. For OP-115 we used three time points: an early time point (day 116 post-infection), a later time point (day 352 post-infection), and one time point when the patient was on anti-retroviral treatment (day 383 post-infection). The frequency of both anti-HERV-K (HML-2) SU and anti-HERV-K (HML-2) TM-specific B cells among the CD19+ population in OP-115 were greater than those observed in the uninfected subject (4.11% vs. 0.73% for SU and 3.46% vs. 0.44% for TM for the early time point) (Figure 4A). However, in OP-115, the frequency of anti-HERV-K (HML-2) SU B cells remained stable over time, while the frequency of anti-HERV-K (HML-2) TM B cells increased with sustained high viral load, before decreasing during antiretroviral therapy, with 3.46% of specific B-cells at day 116, 5.31% at day 352 and 3.62% at day 383 (Figure 4A). Using membrane IgD and CD27 expression to distinguish the maturity of the anti-HERV-K (HML-2) SU and anti-HERV-K (HML-2) TM-specific B cells, we observed that the anti-HERV-K (HML-2) TM- and -SU-specific B cells had similar status at day 116 (Figure 4B). However, as the frequency of HERV-K (HML-2) TM-specific B cells increased over time, the subset of unswitched and switched memory B cells also increased two fold (Figure 4B). During antiretroviral therapy, the frequency of the specific anti-HERV-K (HML-2) TM B cells and the percentage of memory cells decreased to baseline post-infection level, on par with the anti-HERV-K (HML-2) TM antibody response observed in HAART treated patients. These results corroborate the serological studies, and demonstrate the induction of both anti-HERV-K (HML-2) TM B cells and anti-HERV-K (HML-2) TM antibody responses after HIV-1 infection, which decrease in frequency and titer respectively with successful antiretroviral therapy.

### A longitudinal study of anti-HERV-K (HML-2) humoral response before and after HIV-1 infection

**Figure 5:**
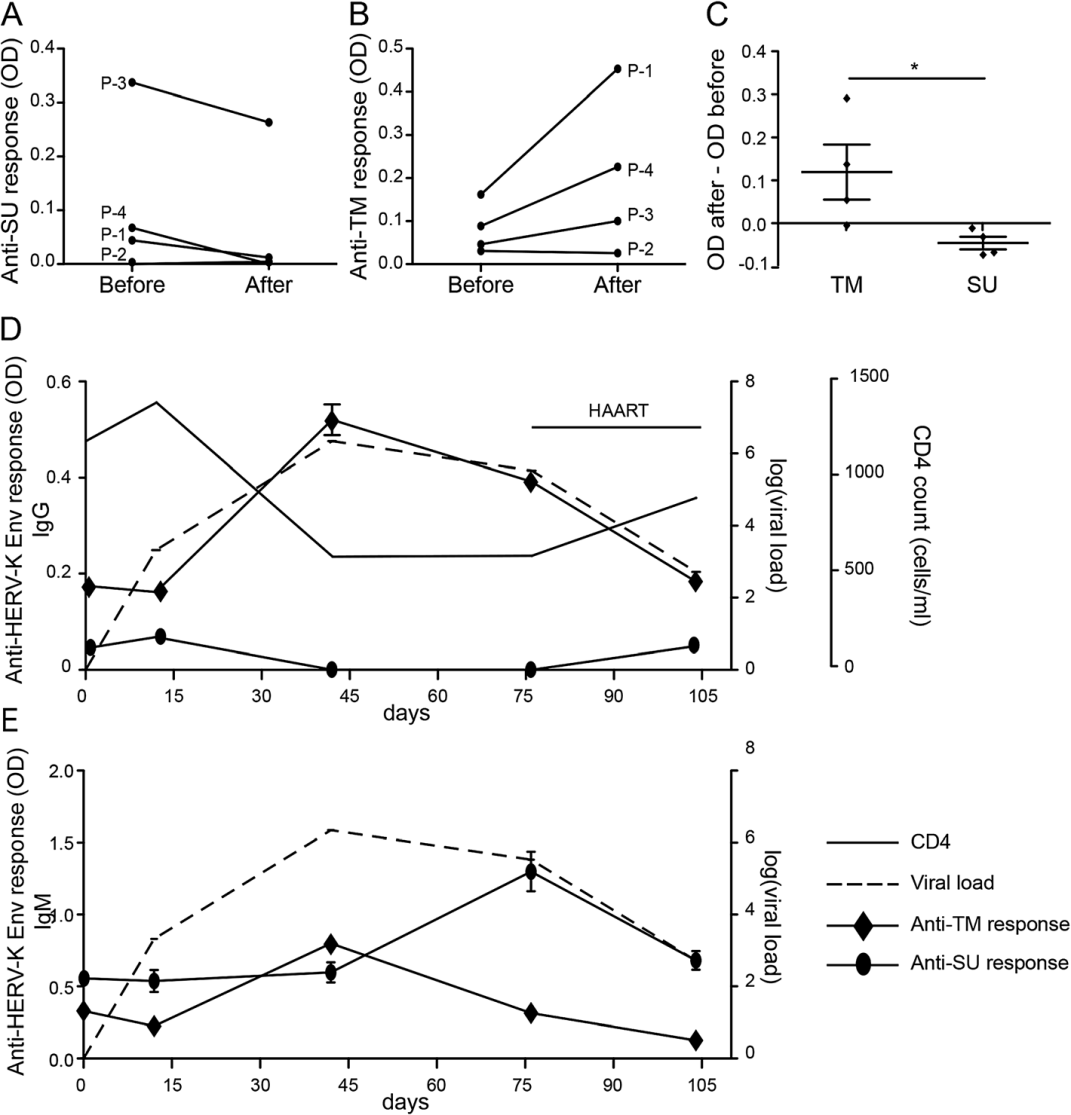
**Kinetics of anti-HERV-K Env antibodies after HIV-1 infection. A,B)** Comparison of the anti-SU **(A)** and anti-TM **(B)** response before and after HIV-1 infection in 4 patients. The time between the two time points did not exceed 1 month. A 1:200 sera dilution was used for this assay. **C)** The evaluation of the kinetics of the response was determined by the subtraction of the OD of the time point after infection by the OD of the time point before infection. A negative result meant a decrease of the response while a positive result meant an increased response. **D,E)** Total IgG **(D)** or IgM **(E)** anti-HERV-K TM and SU were assayed by ELISA on the same plate for 5 time points (1:200 dilution) for the patient OP-1830. The thin black line represents the CD4+ T cell count (cells/mm3) and the hatched line the log10 of the HIV-1 copy number/mm^3^. Statistical analyses were performed using the Mann & Whitney *t*-test; *p < 0.05. Results are typical of three independent experiments.

To better understand the effect of HIV-1 infection on HERV-K (HML-2) Env antibody responses, we monitored the anti-SU and anti-TM antibody responses in four patients in whom samples were available before and after HIV-1 infection. We found the anti-SU response decreased or remained undetectable after infection (Figure 5A) whilst the anti-TM response increased or remained stable (Figure 5B). The mean difference of antibody titer (after infection – before infection) showed a significant difference between TM and SU (Figure 5C) and confirmed the results of the cross-sectional study. These two anti-HERV-K (HML-2) Env antibody responses are differentially modulated during HIV-1 infection, and this occurs from the earliest stage of infection. We then monitored one subject (OP-1830) before and during acute infection, and after antiviral treatment. This subject had a rapid rise in HIV-1 plasma viremia which peaked at day 42, followed by a decrease when the subject started antiretroviral drug therapy at day 76 (Figure 5D). Before infection (day 0 on the graph), the subject had low anti-HERV-K (HML-2) SU and -TM antibody titers. These levels remained static at the time of the first detection of HIV-1 plasma viremia, estimated to be day 10 after infection (Figure 5D). At day 42, the anti-HERV-K (HML-2) TM response increased in parallel with HIV-1 plasma viremia, while the level of anti-SU antibodies decreased below the level of detection. After initiation of antiretroviral treatment, the anti-HERV-K (HML-2) TM response decreased in parallel with the decay of plasma viremia, but the anti-HERV-K (HML-2) SU response reappeared to reach the pre-infection titer, corroborating our previous observation (Figure 2E,F and Figure 4A,B). We observed that the anti-HERV-K (HML-2) TM IgM peaked at the zenith of HIV-1 viremia at day 42 while the anti-HERV-K (HML-2) SU IgM peak was detected after the treatment initiation, at day 76. These observations suggested that the TM response is tightly associated to active HIV-1 replication. Furthermore, the induction of anti-HERV-K (HML-2) TM IgM response during the peak of HIV-1 viremia suggested that HERV-K (HML-2) Env TM protein is preferentially expressed during infection.

### Evidence of HERV-K (HML-2) envelope TM trans-activation and post-transcriptional maturation

**Figure 6:**
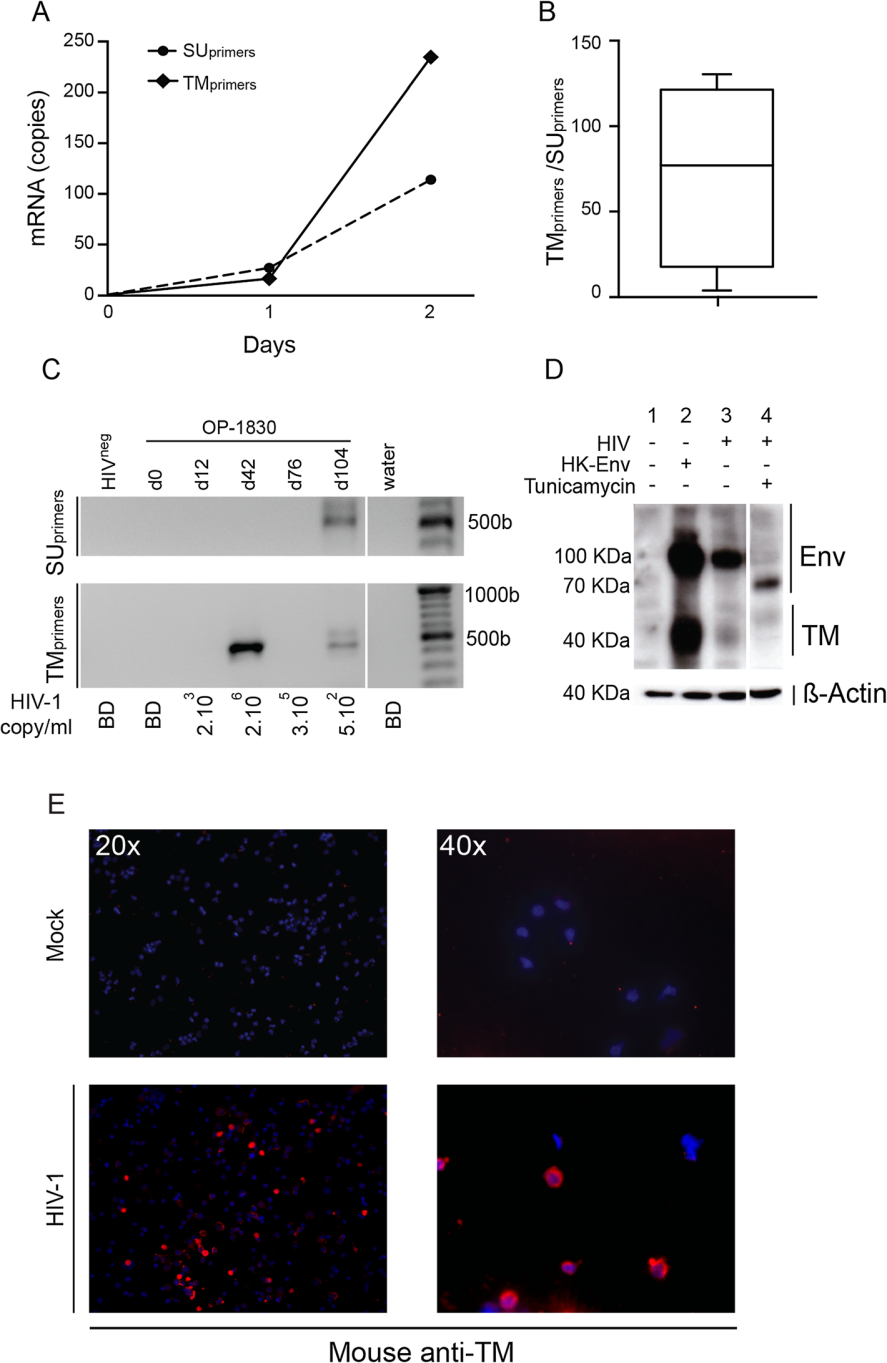
**Evidence of trans-activation and post-transcriptional modification of HERV-K (HML-2) Env TM. A)** HERV-K (HML-2) Env mRNA expression was detected using primers designed to bind the SU domain or TM domain (SU_primers_ or TM_primers_ respectively) at 0,1 and 2 days post infection. Copies of HERV-K (HML-2) Env detected by TM_primers_ (plain line) increased by the time of infection compared to the copies detected by SU_primers_ (dashed line). The graph is a representative experiment of 3 individual independent experiments. Copy number was determined as described previously, and β-Actin was used as reference gene [52]. **B)** The graph represents cumulative data from 3 independent experiments and shows the ratio of copies of HERV-K (HML-2) Env detected by TM_primers_/copies detected by SU_primers_ from HIV-1_LAI_ infected PBMCs 2 days post-infection. Similar data were obtained using primary isolates (91US_4/R5 tropic and BK132/X4 tropic). **C)** Nested PCR. The figure shows the amplicons obtained after the second round of PCR (around 500pb). TM mRNA is over-expressed during the peak of viremia at d42. Antiviral treatment induced the transcription of both SU and TM mRNA at a similar level. HIV^neg^: HIV-1 seronegative low risk donor; OP-1830: HIV-1 seroconverter patient (d0: before infection; d12, d42, d76: after infection without treatment, d104: after treatment); water: the non-template well. **(D)** Assumed precursor proteins at 75 to 90 kDa and TM subunits at 32 to 38 kDa are visible. Hela-T4 cells are infected by HIV-1LAI in presence (4) or not (3) of 10 μl/ml of tunicamycin. HERK-Env transfected cells (2) were used as positive controls for HERV-K TM expression. Uninfected-untransfected cells were used as control for endogenous HERV-K basal expression (1). **(E)** Representative images of HERV-K TM extracellular expression on PBMCs. HERM-1811-5 (anti-TM) mouse monoclonal antibody and goat anti-mouse Alexa555 (red) were used to detect extracellular expression of HERV-K (HML-2) TM.

To investigate whether HIV-1 modified HERV-K (HML-2) Env TM or SU protein expression, we designed primers and probes to detect HERV-K (HML-2) Env transcripts, with one pair of primers amplifying a domain coding for the HERV-K (HML-2) SU epitope (SU_primers_), and another amplifying a domain coding for the HERV-K (HML-2) TM epitope (TM_primers_). We measured HERV-K (HML-2) Env mRNA expression *in vitro* in a time course experiment at d0, d1 and d2 after HIV-1 infection of PBMCs. The expression of HERV-K (HML-2) Env was compared to β-actin mRNA expression. At d0, no HERV-K (HML-2) mRNA expression was detected. At d1, HERV-K (HML-2) Env transcripts were detected using the two pairs of primers (SU_primers_ and TM_primers_) at a similar level. At d2, we observed an increased of transcription of HERV-K (HML-2) Env, but the amplicons detected by the TM_primers_ were overrepresented compared to the transcripts detected by SU_primers_ (Figure 6A). Cumulative data obtained from independent experiments showed that the quantity of transcripts detected using TM_primers_ was significantly superior (mean fold of 77 [4–130]) (Figure 6B). These *in vitro* experiments suggest that HERV-K (HML-2) Env mRNA is expressed under a truncated or mutated form in HIV-1 infected cells.

We then measured HERV-K (HML-2) Env expression using different primers designed for nested PCR amplifying domains coding for either SU or TM in plasma before and after HIV-1 infection in subject OP-1830. Nested PCR was performed on plasma-isolated viral RNA. There was a strong signal at day 42 (peak of HIV-1 viremia and anti-TM IgM) when TM_primers_ were used, while no HERV-K (HML-2) Env expression was detected using SU primers (Figure 6C). At d104, after treatment initiation and decrease in HIV-1 plasma viral load, HERV-K (HML-2) Env mRNA expression was detected with either TM or SU primers (Figure 6A). These results corroborated the *in vitro* results and suggested that the mechanisms involved in the modification of HERV-K (HML-2) mRNA expression *in vitro* occurred *in vivo* as well. These results suggest that HIV-1 induces HERV-K (HML-2) TM protein expression in infected cells.

To investigate HERV-K (HML-2) Env proteins expression in HIV-1-infected cells, we used Hela-T4 cells that are permissive to HIV-1. A commercially available antibody, previously described as detecting the precursor protein (70-100 KDa) and the mature form (about 40 KDa) of TM [32,33] was used to follow HERV-K (HML-2) Env protein expression (Figure 6D). Uninfected cells, used as control for basal protein expression, showed not TM expression (column 1, Figure 6D). Transfected cells with a plasmid coding for the whole HERV-K (HML-2) envelope were used as positive controls (column 2, Figure 6D). HIV-1 infection induced HERV-K (HML-2) Env precursor and, at a lower level, TM protein expression (column 3, Figure 6B). Using the N-glycosylation inhibitor Tunicamycin, we observed that the HERV-K (HML-2) Env protein expressed after HIV-1 infection had a lower MW and inhibited the expression of the mature form of TM (column 4, Figure 6B). These results indicate that HIV-1 infection induces the expression of a fully N-glycosylated HERV-K (HML-2) Env precursor protein and the transmembrane glycoprotein.

A study which reconstituted a functional TM from a consensus HERV-K sequence showed that the N-glycosylation state affects the membrane location and function of TM [32]. Using immunofluorescence on non-permeabilized cells we confirmed the expression of HERV-K (HML-2) Env TM on the cell surface of infected PBMCs (Figure 6E). Taken together, these data support the hypothesis that HIV-1 induces HERV-K (HML-2) Env TM expression on the cell surface.

## Discussion

In this study we investigated how infection with HIV-1 influences HERV-K (HML-2) Env mRNA and protein expression, and assessed the antibody response to the two HERV-K (HML-2) Env subunits, the surface unit (SU) and the transmembrane (TM) protein. We found discordant antibody responses against the two HERV-K (HML-2) envelope protein domains, SU and TM, and determined that HIV-1 influence was at the level of mRNA expression. We showed that HIV-1 infected elite controllers had higher titers of anti-HERV-K (HML-2) TM antibodies, compared to HIV-1 infected patients on effective HAART or healthy uninfected donors. These findings suggest that the anti-HERV-K (HML-2) TM humoral response may play a role in HIV-1 pathogenesis.

The presence and quantity of the antibody response against HERV-K (HML-2) during HIV-1 infection is controversial [30]. Some studies have shown an increase of antibodies against HERV-K (HML-2) [34,35], while others saw no differences between HIV-1-infected and non-infected patients [20,36,37]. We had different results from others [34,35], which might be due to technical differences in methods. The first study describing an anti-HERV-K (HML-2) antibody response showed no difference between HIV-1 infected and uninfected patients [37]. They assessed the antibody response by western blot, detecting sera positive for recombinant SU. They found fifteen percent of HIV-1 infected patients were seropositive for SU, similar to our findings. Another study mapped the anti-SU response in healthy subjects and patients with autoimmune disorders and found a similar epitope on SU [9] (Additional file 1: Figure S1A). In one study, an anti-HERV-K (HML-2) TM antibody had been generated after immunization with a recombinant TM protein, and was directed to a similar epitope present on the ectodomain [33]. This observation, and the mapping obtained in a preceding study [9], agree with our findings that the antibody responses against HERV-K (HML-2) SU and TM are restricted to one peptide for each subunit.

We determined that the origin of the anti-HERV-K (HML-2) TM B-cell and humoral response during HIV-1 infection was the induction of the extracellular expression of the gp36, the transmembrane HERV-K (HML-2) envelope protein subunit. We showed that *in vitro* HIV-1 infection led to the expression of the glycosylated HERV-K (HML-2) Env precursor. The state of glycosylation is crucial for the production of a functional glycoprotein HERV-K (HML-2) Env TM gp36, and a previous study had shown that fully glycosylated gp36 could reach the cellular membrane [32].

HERV-K (HML-2) SU and TM proteins are translated from a unique mRNA coding for the protein precursor. The detection of this unique mRNA using either SU_primers_ or TM_primers_ by qPCR gave non-concordant results. This could be explained by a difference of the primers binding efficiency. Similar number of HERV-K (HML-2) Env mRNA copies were detected using TM_primers_ or SU_primers_ at d1 post-infection *in vitro* and, *in vivo* with different set of TM_primers_ and SU_primers_, when the HIV-1 viral activity was suppressed by the treatment. This suggests that TM_primers_ and SU_primers_ have equivalent efficiency. The increase of HERV-K (HML-2) Env mRNA copies detected by TM_primers_ only at d2 post-infection *in vitro* and d42 at the peak of viremia *in vivo* strongly suggests that an aberrant or truncated mRNA is generated during high HIV-1 transcriptional activity. It is well described that HIV-1 or lentiviral vector integration induces alternative splicing and aberrant transcripts [38-40]. Comparative analysis of transcritomic profiles between HIV-1 infected and uninfected primary T cells revealed that a large number of host genes is virus-induced [38]. These virus-induced sequences are not restricted to coding mRNA but also affect non-coding mRNA [41]. Using next-generation sequencing, it has been determined that the number of altered host gene copies is low at 12 hours post-infection, but dramatically increases at the peak of HIV-1 transcriptional activity after 24 hours post-infection *in vitro*[41]. The chronology of HIV-1-induced host gene transcription supports our finding that the induction of an alternative HERV-K (HML-2) Env mRNA, undetectable with SU_primers_, occurs at the peak of HIV-1 transcriptional activity. The anti-SU antibody response observed in different pathological contexts, such as cancers, is often associated and correlated with an increase in SU protein expression (reviewed in [42]). In our study, our results suggest that HERV-K (HML-2) SU protein expression is poorly induced, because of the low mRNA expression induced by HIV-1. That would explain why we, and others, did not detect an increase in anti-HERV-K (HML-2) SU IgG titer. An alternative HERV-K (HML-2) Env mRNA may code for a dysfunctional SU protein that could be quickly degraded. This hypothesis would support the induction of a specific anti-SU IgM and B-cell responses, but the low level of protein induction would not be enough to induce and maintain a long lasting IgG response.

Although the presence of viral mRNA in the blood of patients suggests the release of viral particles by infected cells [28,43], the absence of a functional HERV-K (HML-2) SU protein makes the release of infectious HERV-K (HML-2) particles unlikely. However, we cannot exclude HERV-K (HML-2) mRNA inclusion in HIV-1 viral particles.

The amino acid sequence we identified as DWNTS (in which the N residue is known to be glycosylated [32]) is nestled between the two cysteines flanking the ectodomain (Additional file 1: Figure S1). This sequence is conserved among HERV-K families (Additional file 1: Figure S1), and is recognized by sera independent of disease stage. There is some sequence homology between the HERV-K (HML-2) TM epitope and an HCV polyprotein (Additional file 1: Figure S1). However, we found no significant difference between HIV-1 positive HCV positive or HIV-1 positive HCV negative subjects in their anti-HERV-K (HML-2) TM response (Additional file 2: Figure S2). The SU epitope also shares some limited homology with the HIV-1 Tat protein (Additional file 1: Figure S1), but the absence of significant increase of anti-HERV-K (HML-2) SU response in HIV-1 infected patients or controllers suggests a limited impact of any potential cross-reactive anti-Tat activity.

To understand the potential role of HERV-K (HML-2) during HIV-1 disease progression, we compared anti-HERV-K (HML-2) TM responses in elite controllers and individuals on effective antiretroviral therapy. Although the level of viremia in these two groups are low, the level of residual HIV-1 replication (as compared to simply production) is known to be higher in controllers than antiretroviral drug treated subjects. We found higher anti-HERV-K (HML-2) TM antibody responses in controllers, suggesting ongoing rounds of HIV-1 replication drive HERV-K (HML-2) expression. An alternative explanation is that a higher level of immune activation in elite controllers compared to antiretroviral-treated subjects or healthy donors, might drive antibody production independent of the level of HERV-K protein production, although as we observed no differences in SU titer this explanation is unlikely. The ratio of anti-HERV-K (HML-2) TM/total IgG and anti-SU confirmed the cross-sectional study, and showed that the TM humoral response was not increased due to polyclonal expansion. Thus, elite controllers have anti-HERV-K (HML-2) TM antibody production even in the absence of detectable viremia (Additional file 3: Figure S3).

Although the only prior study that demonstrated TM protein expression was during pregnancy in the extravillous cytotrophoblast, TM seems to be absent from the cellular membrane at a normal physiological state [33,42]. This raises the possibility that HERV-K (HML-2) may prove to be a marker of an HIV-1 infected cell, and thus could be considered as an HIV-1-Associated Neo Antigen (HANA).

In assessing a potential role for anti-HERV-K (HML-2) TM antibodies in pathogenesis, we observed a correlation between the anti-HERV-K (HML-2) TM response and loss of CD4+ T cells for viremic non-controllers suggesting that during viremic rebound anti-TM antibodies could target HERV-K (HML-2) Env TM-expressing HIV-1 infected CD4+ cells and accelerate CD4+ T cell depletion through mechanisms such as antibody-dependent cell-mediated cytotoxicity or complement-dependent cytotoxicity. However, the presence of an intermediate high anti-HERV-K (HML-2) TM level in elite controllers in the absence of detectable viremia suggests that the antibody response could also play a role in the control of virus through similar mechanisms.

The expression of the HERV-K (HML-2) Env TM protein during HIV-1 infection could have a direct role in immunopathogenesis. Some studies have shown that retroviral transmembrane glycoproteins such as HERV-K (HML-2) TM contain an immunosuppressive domain that inhibits lymphocyte proliferation and plays a role in the immune escape [33,42,44-47]. The increase of expression in viral non-controllers might be linked to progression of the disease. Antibodies present in elite controllers might help to inhibit a TM immunosuppressive effect.

The use of HERV-K (HML-2) proteins as tumor- or viral-associated antigens has already been investigated in different models. HERV-K (HML-2) Env elicits antibodies in patients with breast cancer [19,24] and melanoma [21]. A mouse monoclonal antibody directed against SU showed strong anti-tumor activity *in vitro* and *in vivo* in a mouse model [25,45,48]. In melanoma, pancreatic or prostate cancer, the tumor-associated antigen HERV-K-MEL has been proposed as a specific target for tumor cells [16,23,49]. One concern with a HERV-based approach to vaccination or therapeutic treatment is autoimmunity and immunopathogenesis. However, we have demonstrated the immunogenicity and safety of an endogenous retrovirus vaccine in non-human primates. The vaccine, a combination of DNA and adenovirus coding for Simian-ERV-K, induced specific T-cell and B-cell responses, including an anti-TM antibody response [50]. Thus, ERV-K Env-specific antibodies may not be inherently detrimental to the host due to the restricted expression of ERV-K Env.

## Conclusions

In summary, we have found a novel HERV-K (HML-2) Envelope TM neo-antigen over-expressed in the context of an HIV-1 infected cell, and this stimulates a specific antibody response against HERV-K (HML-2) Envelope TM. Our determination that naturally aviremic HIV-1 infected “elite” controllers have higher titers of this antibody compared to HAART-treated aviremic patients suggests that this antibody response may be of importance in viral control. We speculate that anti-HERV (HML-2) Env TM antibodies could also target infected HIV-1 infected latent “reservoir” cells. As this epitope is highly conserved and not subject to mutation, these findings could lead to a new approach to HIV-1 vaccines or immunotherapy.

## Methods

### Study populations

Samples of peripheral blood mononuclear cells (PBMCs) were selected from participants in two different San Francisco-based HIV-1-infected cohorts: OPTIONS (n = 5) and SCOPE (n = 120). Samples from HIV-1-negative controls were obtained from the Blood Center of the Pacific of San Francisco (n = 80). The study was approved by the local institutional review board (University of California San Francisco Committee on Human Research), research conducted according to the Declaration of Helsinki, and individuals gave written informed consent. Studies were performed on cryopreserved PBMCs and sera.

PBMC and sera samples were obtained from the following categories of chronically HIV-1-infected individuals: 40 elite controllers (EC: naive for treatment, undetectable viral load for two years, CD4 > 350); 40 highly active antiretroviral therapy (HAART: Viremic suppressed with undetectable viral load for at least two years, CD4 > 350), and 40 untreated virologic non-controllers (naive for treatment, viral load >2000 copies/mL).

### HIV-1_LAI_ stock virus

Stocks of HIV-1_LAI_, a CXCR4-tropic laboratory strain, were obtained from the AIDS Research and Reference Reagent Program and amplified on stimulated PBMCs for 7 days [51,52]. HIV-1-infected cells were pelleted at 3,000 rpm for 20 min, and supernatant fluid was passed through a 0.2-μm filter and frozen in aliquots at -80°C. The titers of stocks were determined using TZM cells [53-56].

### *Ex vivo* mRNA isolation nested RT-PCR

Viral mRNA was isolated from 140 μL of plasma using QIAamp® Viral RNA Mini Kit from Qiagen. mRNA obtained were directly used in One-Step RT-PCR Kit (Qiagen) according to the manufacturer. Briefly, 5 μl of viral RNA equivalent to 15 μl of plasma was reverse transcribed at 50°C for 30 min. The 1st round PCR was performed in 20 cycles consisting of 94°C for 30 sec; an annealing step at 55°C for both pairs of outer primers (SU Forward: GTATCAATGGTGGTAAGTCTCC; SU Reverse: CACTGCAATTAAAGTAAAAAT; TM Forward: GCCATTTTATACTRTCGTCCTAA; TM reverse: GACAAAACCRCCATCGTACTCAT) for 30 sec; and an extension step of 90 sec at 72°C. PCR product were next diluted at 1:50 and used as template for the 2nd round PCR. The second round was performed using Phusion® High-Fidelity PCR Master Mix in 35 cycles consisting of 98°C for 10 sec; an annealing step at 60°C for both pairs of inner primers (SU Forward: TGGATAATCCTATAGAARTAT; SU Reverse: TATGTTTGTCTAAACTTTCTGT; TM Forward: GCTGTAGCAGGAGTTGCATTG; TM reverse: TAATTGTAGTACTTCCAATGGTC) for 30 sec; and an extension step of 60 sec at 72°C. PCR products were separated on 1% agarose gels.

#### *In vitro* mRNA isolation and Q-PCR

mRNA was isolated from PBMCs using RNeasy Mini Kit (Quiagen) with on-column DNAase treatment (Qiagen RNase-Free DNase Set) and eluted in 30 μl of RNase-free water according to the manufacturer. A second step of DNAse treatment using TURBO™ DNase (Life Technologies) was performed to eliminate efficiently DNA contaminants. qPCR was performed using TaqMan® One-Step RT-PCR or TaqMan® Universal PCR Mastermix for no-RT control. PCR was performed with one step at 48°C for 30 minutes, 95°C for 10 minutes and 40 cycles consisting of 95°C for 15 sec; an annealing/extension step at 60°C for both pairs of primers (SU Forward CCTGCAGTCCAAAATTGGTT; SU Reverse GCCACACATTCTTCCCAAAC; SU Probe CTCAGGCCACGGGTAAATTA; TM Forward GTTGCGTAAAGCCCCCTTAT; TM Reverse CCCTCTCTTGCTCTCACCAG; TM Probe AATTGGCAACACCGTATTCTG; β-Actin Forward GAGCGCGGCTACAGCTT; β-Actin Reverse TCCTTAATGTCACGCACGATTT; β-Actin PROBE ACCACCACGGCCGAGCGG) for 60 sec. Thermal cycling was performed using a StepOne™ Real-Time PCR System (Applied Biosystems). Data was analyzed using StepOne™ Software (Applied Biosystems). Gene expression and fold induction was determined using the comparative Ct method [57].

#### Plasmid and recombinant proteins

HERV-K (HML-2) Env nucleotide sequence of HERV-K (HML-2) 102 was cloned in a pCDNA3.1 plasmid by CellFree Sciences Co. (Japan). The sequence presents high homology (>98%) with the major HERV-K (HML-2) family members as presented in Additional file 1: Figure S1.

SU and TM sequences were cloned in a pGAEx vector (GENEART vector). Both proteins were produced in HEK293 cells and purified 6 days post transfection by Ni-HiTrap columns by GENEART, Burlingame, CA.

### Transfection and HIV-1-_LAI_ infection/N-glycosylation inhibition

Hela-T4 [58] were plated in 12 well plates at 0.8 × 10^6^ cells/well and transfected with HERV-K (HML-2) Env coding plasmid using Lipofectamine™ 2000 (Invitrogen) according to the manufacturer’s protocol. Briefly, plasmid and Lipofectamin™ were mixed at a 1:2 ratio for 20 min at RT, and incubated with cells for 16 h. Untransfected and transfected cells were washed with PBS and infected with HIV-_LAI_ with 10 μg/ml of tunicamycin (Sigma) or grow medium for 16 h.

### Western blot

Hela T4 cells were lysed in presence of anti-protease cocktail (Sigma) in n-Dodecyl β-D maltoside (Sigma) diluted at 0.1 mg/ml according the manufacturer protocol in dH2O/0.05 M-TRIS HCl/0.15 M-NaCl lysis buffer. Prior to loading, the samples were mixed with lamely buffer 2× and boiled at 95°C for 5 minutes. Approximately, 25 μg of total proteins assayed with BCA Proteins assay kit (Thermo Scientific) were loaded on 4-16% gradient precast gels (Pierce). PVDF (Biorad) membranes were blocked 1 h at RT in PBS/0.05%-Tween 20/10%-non fat dry milk and incubated with mouse monoclonal anti-HERV-K TM HERM-1811-5 (Austral Biologicals) in PBS/0.05%-Tween 20/5%-nonfat dry milk at 1/1000 over-night at 4°C. Membranes were then washed 3 times in PBS/0.1%-Tween 20 and incubated with an HRP-conjugated anti-mouse (Abcam) 2 hours at room temperature. After 6 washes, the membranes were incubated with ECL-plus (GE Healthcare) and exposed at different time points on Kodak® Biomax™ MR film.

### PBMC infection

Fresh PBMCs were isolated by standard Ficoll-Hypaque density gradient centrifugation on fresh blood samples and immediately cryopreserved in fetal calf serum (HyClone, Logan, UT) containing 10% DMSO (Sigma Aldrich, St. Louis, MO) in liquid nitrogen. PBMCs were stimulated with 2 μg/ml of phytohemagglutinin (PHA-L; Sigma, St. Louis, MO) for 48 h in RPMI-10% FBS complemented with IL-2 70U/ml before the addition of HIV-1_LAI_ at a MOI of 0.005 in RPMI-10% FBS.

### Immunofluorescence

PBMCs were fixed in PBS/PFA 2% (Electron Microscopy Sciences) and coated on slides using Cytospin (300 rpm 2 minutes), washed twice with PBS and blocked 15 minutes in PBS/0.5%-BSA/10%-Goat serum. Cells were incubated with mouse anti-HERV-K TM (HERM-1811-5, Austral Biologicals) at 1:50, dilution in a humidified dark room at room temperature for 1 hour and then washed 5 times with PBS. Then, the cells were incubated with an Alexa-555 anti-mouse IgG (Invitrogen) at 1:500 in a humidified dark room at room temperature for 1 hour and then washed 5 times with PBS. DNA was then stained with DAPI (0.5 μg/ml) for 5 minute at RT and the cells washed 5 times in PBS and 1 time with dH20. Coverslips are next mounted with PermaFluor (Thermo Electron Corporation) on microscope slides (Fischerbrand) and dried in the dark. Slides were analyzed on a LEICA DM6000B microscope and photos acquired on Image-pro 6.2 (Scientific Computing).

### ELISA

A set of 172 overlapping “15-mer” HERV-K (HML-2) Env peptides (JPT Peptide Technologies, Berlin, Germany) were used to comprehensively map the antibody response. Positive signals were confirmed by peptides produced by two other companies (New England Peptide and Gene Script). Two peptides corresponding to the immuno-dominant epitopes defined on SU (RPKGKPCPKEIPKES) and TM (HRFQLQCDWNTSDFC) were used for the whole study (Gene Script). 96 microtiter wells plate (Nunc-Immuno Plate MaxiSorp Surface) were coated for 1 hour at 37°C with peptides at 10 μg/ml in PBS or over-night at 4°C with recombinant protein (GeneArt) at 5 μg/ml in PBS. Plates were then washed 3 times with 200 μL of PBS/0.05%-Tween 20 and blocked with 100 μL of blocking buffer (PBS/2.5%-BSA) at room temperature (RT). The samples were diluted in blocking buffer and incubated 2 h at RT in duplicates. Plates were then washed 3 times with 200 μL of PBS/0.05%-Tween 20. An anti-human IgG or anti-human IgM HRP-conjugated secondary antibody was diluted at 1:1000 in blocking buffer and incubated at RT for 1 hour. Plates were then washed 6 times with 200 μL of PBS/0.05%-Tween 20 and incubated for 10 minutes with 100 μL of TMB (Invitrogen). Addition of 50 μL H2SO4 2 M stopped the reaction. The plates were read at 450 nm and 690 nm for the background on a plate reader. Background from 690 nm uncoated wells and PBS-BSA as negative controls was subtracted from the mean absorbance of the coated wells. Titers were determined as the reverse of the highest dilution of each serum sample that gives an optical density superior than its respective negative control average. Detection of anti-SU antibodies. ODs were normalized with serum from a high responder in a standard curve. The STDEV intra experiment was less than 7%. Detection of anti-TM antibodies. Sera were used at 1:400. OD were normalized with serum from a high responder in a standard curve. The STDEV intra experiment was less than 4%.

### Tetramer preparation and B-cell staining

Adapted from Franz et al. [59].

All tetramers were prepared freshly for each experiment. Biotinylated-SU or -TM peptides (Gene Script) were incubated with premium-grade phycoerythrin-labeled streptavidin (Molecular Probes) for at least 20 minutes on ice at a molar ratio of 4:1. Before cell staining, tetramer preparations were centrifuged for 10 minutes at maximum speed to remove aggregates.

PBMCs were isolated by standard Ficoll-Hypaque density gradient centrifugation on fresh blood samples and immediately cryopreserved in fetal calf serum (HyClone, Logan, UT) containing 10% DMSO (Sigma Aldrich, St. Louis, MO) in liquid nitrogen. The cryopreserved cells were stored in liquid nitrogen until they were used.

Cells were thawed, washed, counted, and resuspended in PBS/5% FCS. For memory B-cell labeling, cells were enriched with the use of Human B Cell Enrichment Kit (Miltenyi). After enrichment, cells were adjusted to a density of 5 × 10^6^ cells/mL and stained with SU-Tet or TM-Tet and incubated on ice for 30 minutes with intermittent gentle vortexing. Cells were co-stained with, IgD-APC (BD Biosciences), CD27-PacificBlue (BD Biosciences), and CD19-APC-H7 (BD Biosciences) for an additional 20 minutes on ice. LIVE⁄DEAD® Fixable Dead Cell Stain Kit was used to discriminate live and dead cells. Cells were washed and stored in 2% paraformaldehyde at 4°C until acquisition on the LSR-II flow cytometer.

For all flow cytometry experiments, data were acquired with an LSR-II system (Becton Dickinson). At least 100,000 events were collected and analyzed with FlowJo software, version 9.0 (Tree Star, Ashland, OR).

### Statistical analyses

Humoral responses assayed by ELISA were compared between groups using the Kruskal-Wallis, Dunn’s multiple comparison, or two-tailed Mann–Whitney t tests. Linear regression and Spearman correlation analyses were used to measure associations between humoral response and HIV-1 viral load or CD4+ T cells count. All tests were conducted using GraphPad Prism, version 5.00 (GraphPad Software, San Diego, CA), with the statistical significance of the findings set at a p value of less than 0.05.

## Competing interests

H-AM, SGD, JBS and DFN are listed on a patent application filed by UCSF in respect of this work.

## Authors’ contributions

H-AM designed and performed the experiments and wrote the paper, MM helped design and perform the experiments, DS helped design and perform the experiments, SD helped design the experiments and provided clinical samples, JNM provided clinical samples, JJK helped design the experiments, JBS helped design the experiments and co-wrote the paper, DFN helped design the experiments, and co-wrote the paper. All authors helped edit the paper. All authors read and approved the final manuscript.

## Authors’ information

H-AM and MM are postdoctoral fellows in DFN’s laboratory at UCSF. DS is an Adjunct Assistant professor in DFN’s laboratory. SGD is Professor of Medicine at PHP/AIDS Division, UCSF. JNM is Professor of Epidemiology and Biostatistics, UCSF. CDP is Associate Professor of Medicine at PHP/AIDS Division. FMH is Professor of Medicine at PHP/AIDS Division, UCSF. JBS is Assistant Professor at OSHU. DFN is Adjunct Professor of Medicine, Division of Experimental Medicine, UCSF, and Chair, Department of Microbiology, Immunology & Tropical Medicine at GW University, Washington, DC.

## Supplementary Material

Additional file 1: Figure S1 Epitope mapping and comparison homology studies. A, and B), Identification of the residues recognized by sera on SU and TM peptides. A set of peptides with the single mutation X to A were used in a peptide-based ELISA to determine which amino acids (aa) were recognized. The graphics represent the% of binding of the sera on the mutated peptide compared to the original peptide [(OD mutated peptide/OD original peptide)*100]. The original peptide corresponds to the peptide that gave the better signal for the mapping (see method section). The precise aa sequence was determined by reduction of binding. For both epitopes, sera from infected and healthy donor were used (6 for SU and 9 for TM). The graphs show the average of the 6 and 9 sera used respectively for SU and TM mapping. The results show that the sequences recognized were PKEIPKE for SU epitope and DWNTS for the TM epitope. C) Comparison of the sequence of SU and TM peptides among the different main HERV-K (HML-2) families. Dots represent the same aa as the original sequence; (-) represents an aa deletion; a letter represents a mutation. On the left are the names of the HERV-K (HML-2) families. Corresponding PubMed access numbers: K101 (AF164609.1); K102 (AF164610.1); K103 (AF164611.1); K104 (AF164612.1); K107 (AF164613.1); K108 (AF164614.1); K109 (AF164615.1); K113 (AY037928.1); K115(AY037929.1) D) Major homology of sequence between SU and TM sequence with other viruses. Underscored letters are the epitopes determined in Additional file 1: Figure S1A; homologies are represented in bold. The homologies were determined by using Blast tool from the PubMed website. Click here for file

Additional file 2: Figure S2 Anti-TM response in HCV positive patients. Results from the cross sectional studies were analyzed with respect to the HCV serostatus of the 120 HIV-1 infected patients. 47 were identified as HCV positive and 69 as HCV negative. This clinical information was not available for 4 patients. The statistical significance of data between the two groups was established using the Mann & Whitney *T* test. Click here for file

Additional file 3: Figure S3 Ratio anti-HERV-K (HML-2) Env antibodies/total IgG. The total IgG level was assayed using a human IgG ELISA kit (Mabtech) according to the provider’s protocol. The sera were diluted at 1/4000000. A ratio was calculated as follows: anti-SU or anti-TM titer (OD)/total IgG (OD). n = 40 for each groups. The ratio difference shows the humoral responses against SU and TM do not result from a nonspecific polyclonal expansion. The statistical significance of data between the different groups was established using ANOVA Kruskal-Wallis and Dunn’s Multiple Comparison. The figure shows the representative results of three independent experiments. A p value <0.05 was considered as significant. *p < 0.05, **p < 0.01, ***p < 0.001. Click here for file
